# Translational landscape during seed germination revealed by ribosome profiling

**DOI:** 10.1111/tpj.70663

**Published:** 2026-01-07

**Authors:** Bing Bai, Run Qi, Wei Song, Harm Nijveen, Leónie Bentsink

**Affiliations:** ^1^ Laboratory of Plant Physiology, Wageningen Seed Science Centre Wageningen University Wageningen 6708 PB The Netherlands; ^2^ The Key Laboratory of Watermelon and Cabbage Digital Seed Industry, Ministry of Agriculture and Rural Affairs, P.R. China Ningbo Weimeng Seed Industry Co., Ltd. Ningbo China; ^3^ Bioinformatics Group Wageningen University Wageningen 6708 PB The Netherlands; ^4^ Plant–Microbe Interactions, Department of Biology Utrecht University Utrecht 3508 TB The Netherlands; ^5^ State Key Laboratory of Agricultural and Forestry Biosecurity, College of Plant Protection Nanjing Agricultural University Nanjing China

**Keywords:** seed germination, ribosome profiling, translational control, upstream open reading frame (uORF), long non‐coding RNAs, *Arabidopsis thaliana*

## Abstract

Seed germination is crucial for agricultural reproduction. A deep understanding of this process can secure healthy growth at the early phases of plant development and therefore yield. Recent research indicates that germination is a complex process involving translational regulation. A large group of seed‐stored mRNAs together with newly synthesized transcripts are regulated by post‐transcriptional mechanisms and selectively translated at different stages to support the germination process. To investigate the mechanism of translational control, we performed ribosome profiling on mRNAs of distinct physiological stages during *Arabidopsis thaliana* seed germination. The presence of ribosome association on mRNAs with three‐nucleotide periodicity indicates their capacity for translation. Dry seeds, in which translation is on hold, are characterized by a unique ribosome association landscape with a higher ribosome association at the 5′ and 3′ UTR, compared with physiological stages that show active translation. Start codon‐specific stalling of ribosomes in dry seeds is associated with an adenine‐enriched sequence motif. Throughout germination, codons encoding glycine, aspartate, tyrosine, and proline are the most frequent ribosome pausing sites. Moreover, the non‐coding ribosome‐associated RNAs that we identified are indeed translated, as was revealed by investigating total seed proteome data. Seed‐specific upstream open reading frames (uORFs) have been identified that may play a role in translational regulation of early seed germination. Altogether, we present a first ribosome profiling analysis across seed germination that illuminates various regulatory mechanisms that potentially contribute to the seed's survival strategy.

## Significance Statement

We have performed polysome profiling to reveal mechanisms underlying the translational control of seed germination. In dry seeds, we identified ribosome stalling at both the 5′ and 3′ untranslated regions (UTR) and start codon‐specific stalling at adenine‐enriched sequence motifs. Moreover, we have identified a role for seeds specific upstream open reading frames (uORFs) and long non‐coding mRNAs in the regulation of translation during seed germination.

## INTRODUCTION

Seeds are the primary structures responsible for plant propagation and are key for the survival of plants. During germination, the seed embryo develops into a seedling, which subsequently grows into a mature plant. In their fully matured state, seeds store large amounts of mRNA. The translation of these stored mRNAs is essential for germination to take place; translation inhibitors such as cycloheximide can completely inhibit germination while inhibitors of transcription cannot (Rajjou et al., 2004). Moreover, seed‐stored mRNAs can survive for years while still maintaining their capacity for translation (Dure & Waters, 1965; Sano et al., 2016; Waters & Dure, 1966; Yashina et al., 2012). The role of mRNA storage in dry seeds and the regulation of their translation during seed germination is a key question in the field of seed biology. Studies addressing this phenomenon have mostly focused on *Arabidopsis thaliana*. In Arabidopsis, stored mRNAs are predominantly associated with monosomes, single ribosome units, that are not actively involved in translation (Bai et al., 2020). When dry seeds take up water, the translation of monosome‐associated transcripts is initiated. As more ribosomes bind to a single mRNA molecule, a polysome is formed, allowing multiple proteins to be synthesized simultaneously from a single mRNA. This process, which occurs in the first 6 h after the start of seed imbibition, is strongly regulated and referred to as the Hydration Translational Shift. A second translational shift occurs around the moment that the seed embryonic root (radicle) breaks through its surrounding layers, the testa and the endosperm, and is referred to as the germination translational shift (Bai et al., 2017). The translational shifts referred to indicate a change in translational efficiency (TE), e.g. the proportion of ribosome‐bound mRNA over total mRNA, a ratio used to evaluate the extent of translation for specific mRNAs (Ingolia et al., 2009; Juntawong et al., 2014; Liu et al., 2012).

The fate (synthesis, translation or degradation) of ribosomes on a transcript is influenced by interactions between the ribosome and mRNA sequence features such as the 5′ untranslated region (5′ UTR), upstream open reading frames (uORFs) and the 3′ untranslated region (3′ UTR). Therefore, accurately determining the position of the ribosome on stored mRNAs in seeds can provide insights into the state of mRNA storage and their fate during seed germination when the translation is re‐activated. Information about the ribosome position on the mRNA can be revealed by ribosome profiling. Deep sequencing of ribosome‐protected mRNA fragments (ribosome footprint, RP) identifies the position of the ribosome on the RNA molecules. This technique has been extensively used across various species, including yeast, bacteria, *Archaea*, *Drosophila*, and plants, to investigate translational control in response to environmental stimuli and during developmental processes (Gelsinger et al., 2020; Hsu et al., 2016; Ingolia et al., 2009; Zhang et al., 2017). In crop seeds, ribosome profiling has been used to study translational control during seed maturation (Guo et al., 2023) and to study tissue‐specific translation (Zhu et al., 2023).

Sequence features such as the length of the UTRs, GC content, and RNA structure are associated with mRNA TE in Arabidopsis (Bai et al., 2017). Moreover, uORFs that are located in the 5′ UTR of some eukaryotic mRNAs, upstream of the main ORF (mORF), are known to regulate translation. These uORFs can cause stalling or releasing of the ribosome from the mRNA and thereby inhibit translation of the mORF (Meijer & Thomas, 2003; Wiese et al., 2004). The uORFs' translational control mechanism is conserved; across multiple plant species, the interaction between the uORF‐encoded peptides and metabolites, such as sucrose, polyamine, thermospermine, and other metabolites, have been identified (van der Horst et al., 2020).

In addition to mRNAs, eukaryotic cells contain a substantial number of non‐protein coding RNAs. Among these, long non‐coding RNAs (lncRNAs) are defined as transcripts longer than 200 nt that either lack an ORF entirely or do not contain an ORF encoding more than 100 amino acids. lncRNAs are known to play important regulatory roles in plants. For example, some have been shown to encode functional peptides that are associated with plant morphogenesis and stress‐induced plasticity (Bazin et al., 2017; Wang et al., 2019; Yu et al., 2019; Zeng et al., 2018). The majority of lncRNAs are transcribed by RNA Polymerase II and contain typical mRNA‐like features, such as a 5′ m7G cap and a 3′ poly(A) tail. Therefore, lncRNAs are processed in the same way as mRNAs (Wu et al., 2017). lncRNAs are characterized by a lower conservation and abundance, more tissue‐specific expression, and lower splicing efficiency than mRNAs (Ulitsky & Bartel, 2013). Over a thousand lncRNAs are identified as associated with ribosomes, which indicates that they have the potential to be translated (Bazin et al., 2017; Zeng et al., 2018).

To obtain a better understanding about translational regulation during seed germination, we performed ribosome profiling (ribosome footprinting, Ribo‐seq). We characterize the features and dynamics of the ribosome‐associated mRNAs in both dry seeds and during seed germination. Moreover, we utilize transcriptome‐wide ribosome position maps across the course of seed germination to explore the function of uORF‐mediated translational control and to identify lncRNAs with a translation potential.

## RESULTS

### Ribosome profiling during seed germination

**Figure 1:**
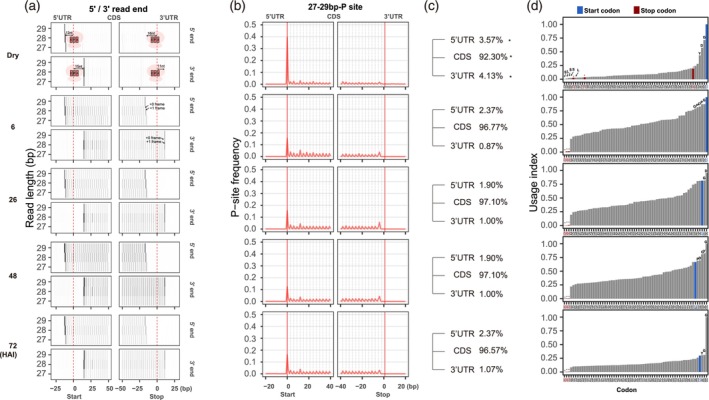
Ribosome dynamics during seed germination. (a) Meta‐gene heatmap of ribosome footprinting around start and stop codons. Heatmaps display aggregate ribosome occupancy based on the 5′ end (top panels) and 3′ end (bottom panels) of ribosome‐protected fragments (RFs), aligned relative to annotated start and stop codons. Signals are shown for RFs of 27, 28 and 29 nt in length across five stages of germination: dry seeds and seeds at 6, 26, 48, and 72 h after imbibition (HAI). For the dry seed panels, a schematic ribosome is superimposed on the plots. The E, P, and A site position and the distance of the P site to the start and stop codon are indicated to illustrate ribosome positioning. (b) Metaprofile of ribosome occupancy displaying the relative abundance of P sites around the start and the stop codon of annotated CDSs (fragment lengths, 27–29 nt combined) during seed germination. (c) The percentage of RFs in the 5′ UTR, CDS, and 3′ UTR at each stage during seed germination. Asterisk indicate the significance comparing percentages in dry seeds to all other stages (*P* < 0.05). (d) Codon usage based on in‐frame P sites during seed germination. The codon usage index is calculated as the frequency of in‐frame P site positions associated with each codon along the CDSs, normalized for codon frequency. For the most frequent codons, their corresponding amino acid is displayed above each bar. The blue and red bars represent the start and stop codons respectively. Note, the AUG codon includes both start codons and internal methionine codons within the CDS. The profiles of one representative biological replicate are presented.

To determine the precise position of ribosomes on mRNAs during translation, ribosomes were isolated during seed germination at 0, 6, 26, 48 and 72 HAI. These time points correspond to the physiological stages: dry seeds, seeds at early imbibition, seeds before testa rupture, seeds at 80% of endosperm rupture and 80% of seedling greening. These stages were shown to exhibit extensive translation dynamics (Bai et al., 2017). Transcriptome‐wide ribosome profiling (Ribo‐seq) was performed at a depth of approximately 100 million reads per sample. Reads mapping to rRNA and tRNA, and unmapped reads were removed. Reads mapping to non‐coding RNAs were stored as a separate dataset for further analyses (Table S1). 8.7% of the total number of reads per sample mapped to mRNA (Table S1). As expected for translating ribosomes, we observed a strong 3‐nt (codon‐sized) periodicity in mapped reads representing RF in the size range between 27‐ and 29‐nt. In dry, 6 and 26 HAI seeds, the 28‐nt RFs are most pronounced, whereas at the later time points during seed germination, the 29‐nt RFs become more pronounced (Figure 1a; Figure S1). The −12‐ and +15‐nt position marked the ribosome extremities at the 5′ and 3′ respectively, at the start codon context (Figure 1a). At the stop codon, in addition to the 3‐nt periodicity, we also observe footprints in the +1 reading frame, both at the 5′ and 3′ ribosome extremities (Figure 1a,b). The ribosome P site serves as a reliable reference point for ribosome positioning, since the start codon is aligned with the P site during translation initiation. To further validate the genuine RFs, the P site position was visualized across the up‐ and downstream sequences of start and stop codon context. Like the ribosome extremity plot, the ribosome P site metaplot also shows a clear periodic distribution (Figure 1b). This periodic P site signal was identified in the CDS at all physiological stages including a strong general peak at the start codon and a smaller peak at the 5th codon position (Figure 1a,b; Table S2), indicative of initiation and post‐initiation translational pausing (Han et al., 2014). As expected, in dry seeds, the peak signal was overall lower compared with the imbibed stages (Figure 1a,b; Table S2), consistent with the repressed translational activity in dry seeds. Strikingly, in dry seeds ~7% of the total mapped reads were positioned at start codon, this corresponds to 40% of the reads that are positioned at the P site of the ribosome in the displayed region of the metaprofile (Figure 1b). This is significantly higher than in the other stages (~1%–2% for 6–72 HAI; *t*‐test, *P* < 0.05, Table S3), corresponding to 10–20% of the reads that are positioned at the P site of the ribosome (Figure 1b). Summing up all the RFs across the transcripts, 92.3% of the RFs in dry seeds mapped to CDSs, 3.57% to 5′ UTRs, and 4.13% to 3′ UTRs, while at other time points approximately 96–98% of the RFs mapped to CDSs, ~1.9–2.3% to 5′ UTRs, and ~0.8–1% to 3′ UTRs (Figure 1c; Tables S2 and S3), indicating a reduced ribosome loading in CDS and an increased loading in both 5′ and 3' UTRs in dry seeds compared with other physiological stages (*P* < 0.05).

The strength of ribosome association can be influenced by the codons encountered during translation. To identify the codon that contributes the most to ribosome pausing (temporary halting of the ribosome during the process of translation), the codon usage index was computed based on the in‐frame P site position (Figure 1d). In dry seeds, ribosomes were paused mainly at the start codon followed by codons encoding glycine (G), aspartate (D), and tyrosine (Y). Interestingly, ribosomes in dry seeds were also partially paused at stop codons, especially at the UAG codon (codon usage index = 0.13, Table S4), which might reflect incomplete translation termination. At 6 HAI, ribosomes were associated most frequently with the start codon followed by codons for glycine (G), proline (P), and aspartate (D), while ribosome occupancy at the stop codon was strongly reduced at this stage. From 26 HAI onwards, as germination progressed and translation became fully active, the start codon was no longer the primary ribosome pausing site. Instead, codons for glycine (G), aspartate (D), tyrosine (Y), and proline (P) emerged as the most frequent pausing positions. No stalling at the stop codon was identified, likely because only the P site position was analyzed. Due to polypeptide chain elimination at the A site, the tRNA encoding the stop codon does not reach the P site. Moreover, ribosome foot printing around the stop codon identified a distinct peak 4 nucleotides upstream of the stop codon (Figure 1b). This feature, which coincides with the previously mentioned +1 shift in the RF around the stop codon (Figure 1a), has been reported previously and was proposed to be necessary for the accurate termination of protein synthesis in eukaryotic cells. Cryo‐electron microscopy analyses in mammals revealed that ribosome complexes containing the release factor (eRF1) interact with the stop codon in the A site; this results in a change in the configuration of the 18S ribosomal RNA which then pulls the fourth position base into the A site (Brown et al., 2015; Heyer & Moore, 2016).

### Translational features of start codon‐enriched genes in dry seeds

**Figure 2:**
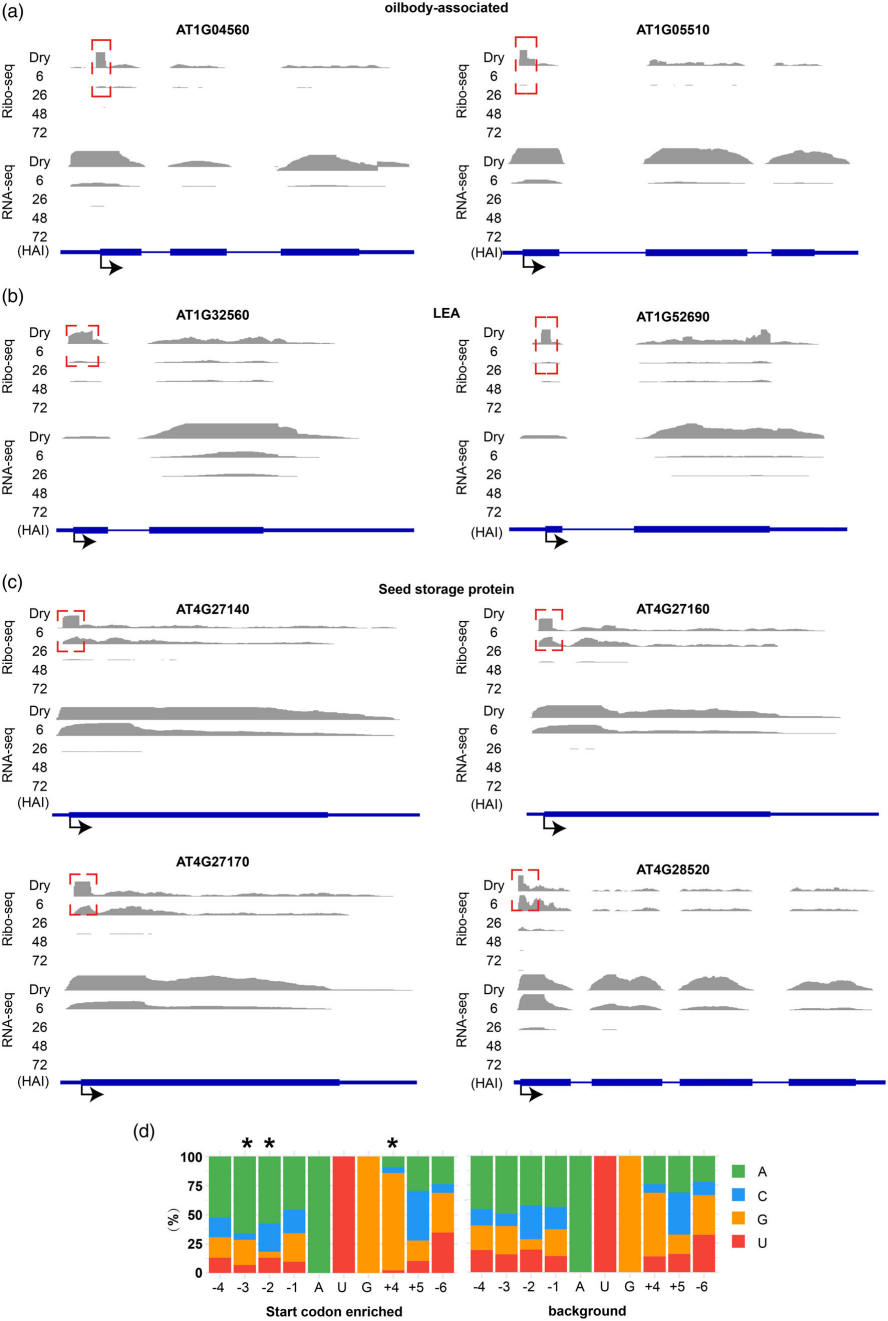
Start codon‐enriched ribosome footprints in dry seeds. (a–c) Coverage plots showing translational (Ribo‐seq) and transcriptional (RNA‐seq) read densities during seed germination for representative genes with start codon‐enriched ribosome footprints in dry seeds and at 6 HAI. Genes are grouped by function: (a) oil body‐associated, (b) late embryo abundant (LEA), and (c) seed storage protein genes. The boxes with the red dashed lines indicate the area at start codon where the reads differ between dry and 6 HAI seeds. Data from three independent biological replicates were pooled, and results are presented using the same read density scale for the compared groups. (d) Start codon context. Distribution of each nucleotide per position relative to the start of the CDS in the start codon‐enriched RF genes compared with the background genes are shown. The asterisk indicates a significant difference in nucleotide distribution at the −3, −2 and +4 positions (*P* < 0.0001, Chi‐squared test).

Dry seeds show a dominant RF peak at the start codon (Figure 1a–c). We selected 176 genes in which at least 10% of the total ribosome occupancy occurs at the start codon (Figure S5c,d; Table S5). In contrast, the transcripts with the proportion CDS RF distribution also show a relatively high start codon proportion in dry seeds compared with other stages (Figure S5e,f). Indicating that dry seeds have a generally high start codon RF association. GO analyses show that these genes are enriched for biological functions with terms such as seed oil body biosynthesis, lipid storage, and seed maturation (Table S6). When investigating the translational behavior of these genes, two distinct groups were identified. The first group consists of genes that are enriched for RFs at the start codon in dry seeds but show a marked depletion at 6 HAI. Examples include genes encoding oil body‐associated proteins and late embryogenesis abundant (LEA) proteins. Their transcripts are known to accumulate during maturation and are likely degraded immediately upon imbibition, consistent with their reduced transcript level during germination (Figure 2a,b). The second group includes genes that also show RF enrichment at the start codon in dry seeds but exhibit increased ribosome occupancy in their CDS upon imbibition (6 HAI). Their transcript abundance reduces sharply upon further imbibition (Figure 2c). These genes include seed storage protein genes. The accumulation of seed storage proteins during seed maturation indicates that the translation of these transcripts starts already during seed maturation and continues during early imbibition. These two modes of ribosome association patterns may represent different fates for seed‐stored mRNAs with varying function during seed germination.

To investigate whether there is a sequence basis for ribosome pausing at the start codon, the sequence context surrounding the AUG start codon of the 176 start codon‐enriched genes was analyzed. This analysis revealed the motif AAAGAUGG (Figure 2d), whose nucleotide composition differs significantly from background sequences at positions −3, −2 and +4 relative to the adenine of the start codon. Specifically, positions −3 and −2 are enriched for adenine, while position +4 shows increased guanine and reduced adenine and uracil (Table S7). This motif closely resembles the canonical Kozak consensus sequence in dicot plants AAAMAAUGGC (where M = A or C) (Joshi et al., 1997).

### Translational changes of mRNAs and lncRNAs during seed germination

Earlier, we have evaluated the TE of mRNAs during seed germination using polysome profiling (Bai et al., 2017). The shift in TE from dry to 6 HAI seeds that we called the “hydration translational shift” (HTS) is also the most significant translational shift present in the Ribo‐seq results of this study (Figure S2a,b; Tables S8–S10). After the 6 HAI time‐point, translational regulation was predominantly negative, with more genes being downregulated than upregulated (Figure S2b). GO analyses revealed that this downregulation involved genes related to photosynthesis, fatty acid biosynthetic processes, cell wall modification, and glucosinolate metabolic processes from 6 to 26 HAI. From 26 to 48 HAI GO categories included rRNA and ribosome biogenesis and from 48 to 72 HAI cellular responses to oxygen levels and immune response. Moreover, the translationally regulated genes displayed diverse patterns of TE changes during seed germination, meaning that they can be caused by the changes in ribosome‐associated mRNA (R), the total RNA (T), or both (additive) (Figure S2c–f). Comparing the differentially expressed genes between consecutive time points, we find a large overlap between the RNA‐seq used in this study and the previously published microarray results, indicating the robustness of cross‐platform translatome profiling during seed germination (Figure S3). At the translational level, the overlap is considerably lower, likely due to methodological differences: the earlier study employed polysome profiling while the Ribo‐seq used here also captures monosomes.

The sequencing‐based method used in this study allowed us to explore ribosome association with non‐coding RNAs such as lncRNAs during seed germination. In total, we detected 1594 and 218 non‐coding RNAs from the RNA‐seq and Ribo‐seq datasets based on genome annotation (Table S11). To further investigate the coding potential of these identified lncRNAs in seeds, both Ribo‐seq and RNA‐seq detected lncRNAs were investigated for the presence of ORFs. These analyses revealed that 463 lncRNAs expressed during seed germination contain a predicted ORF, among which 25 lncRNAs were found to be under translational control at distinct physiological stages (Figure S4; Tables S11 and S12). To validate the translation potential of the ORF containing lncRNAs, the predicted peptide sequences encoded by their ORFs were used for peptide discovery. The proteomic data for these analyses were obtained from a total proteome analysis that was performed on the same germination time points as those analyzed in the present study (Bai et al., 2021). In total, 50 out of 426 lncRNA‐encoded peptides were identified in the seed total proteome during germination (Table S13), providing strong evidence that the ORFs within these lncRNAs are translated and may play a functional role during seed germination. For most of these peptides, only a single unique peptide was identified, which did not allow for correlation analyses between the lncRNA‐encoded peptides and the ribosome‐associated lncRNAs. Such an analysis would have been anyway challenging due to the known delay in protein translation during seed germination. Polysomal mRNA abundance at a germination time point was found to correlate best with protein abundance of the next time point (Bai et al., 2021).

### uORFs impact the translation of main ORFs during seed germination

**Figure 3:**
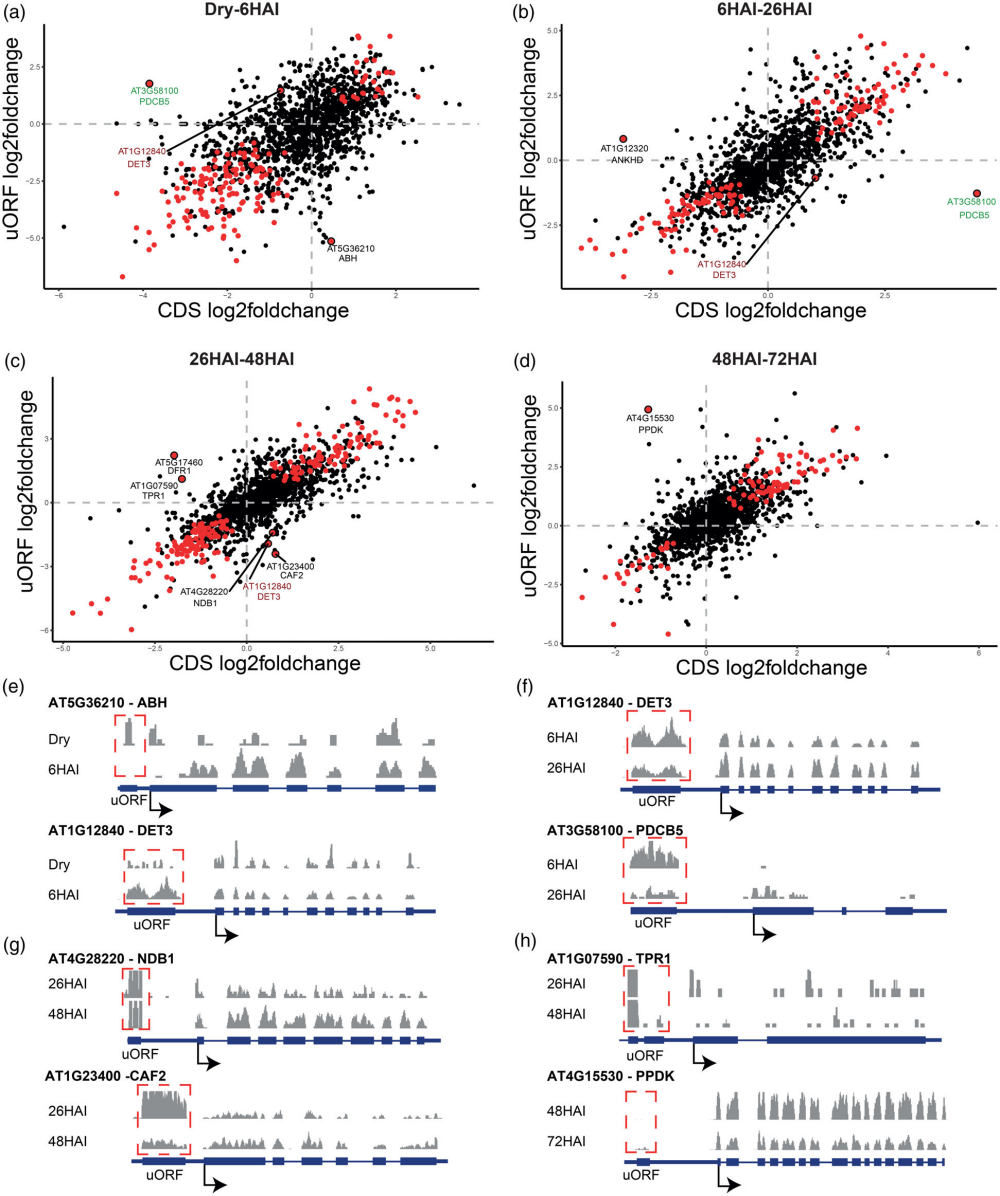
uORF‐related translational changes during seed germination. (a–d) Scatter plots show the log_2_ fold changes in ribosome footprints for the CDS versus the uORF region for ribosome‐associated transcripts across four germination transitions: dry to 6 HAI (a), 6–26 HAI (b), 26–48 HAI (c), and 48–72 HAI (d). Red dots indicate genes with significant changes in ribosome occupancy for the stages that are compared. Genes labeled with AGI codes and gene names are those with significant shifts in ribosome distribution during germination progression. AGI labels colored in red and green represent genes for which the ribosome occupancy changed significantly at multiple transitions. (e–h) RF metaprofiles of genes with either an increase in RF at the uORFs and a decrease at the mORF or vice versa. The red frames indicate the uORF region of each gene. Data from three independent biological replicates were pooled, and results are presented using the same read density scale for the compared groups.

uORFs are known to regulate the translation of their downstream mORF by interrupting ribosome scanning during translation (Meijer & Thomas, 2003; Yamashita et al., 2017). To evaluate whether uORFs influence mORF translation during seed germination, the fold change in RF read counts for uORFs between the two consecutive time points during germination was compared with that of the corresponding mORFs (Figure 3a–d). Globally, RF changes at uORFs agree with the changes at the mORF, suggesting that the majority of genes are not translationally regulated by their uORFs. However, comparison across consecutive physiological stages identified nine genes (FDR < 0.05) that exhibit a negative correlation between uORF and mORF translation, characterized by either an increase in RF at the uORF and a decrease at the mORF or vice versa. These uORF‐regulated genes were *ALPHA/BETA‐HYDROLASES SUPERFAMILY PROTEIN*, *ABH SUPERFAMILY PROTEIN* (*ABH*, AT5G36210), *DE‐ETIOLATED 3*, *DET3* (AT1G12840), *PLASMODESMATA CALLOSE‐BINDING PROTEIN 5* (*PDCB5*, AT3G58100), ankyrin repeat/KH domain protein (*ANKHD*, AT1G12320), *NAD(P)H DEHYDROGENASE B1* (*NDB1*, AT4G28220), *CHLOROPLAST RNA SPLICING2‐ASSOCIATED FACTOR 2* (*CAF2*, AT1G23400), *TETRATRICOPEPTIDE REPEAT (TPR)‐like superfamily protein* (*TPR1*, AT1G07590) and *PYRUVATE ORTHOPHOSPHATE DIKINASE* (*PPDK*, AT4G15530) (Figure 3e–h; Table S14). In dry seeds, the uORF of *ABH* (AT5G36210) was found to be associated with ribosomes. Upon imbibition (6 HAI), the uORF association decreases (*P* = 1.24 × 10^−5^) accompanied by a concurrent increase in mORF ribosome association (*P* = 0.04). This indicates that the ribosome was likely stalled in the dry seeds (Figure 3e; Table S13). In contrast, *DET3* (AT1G12840) ribosome association was relatively low in dry seeds, but substantially increased at 6 HAI (*P* = 4.48 × 10^−5^), which resulted in a decrease of RFs in the mORF (*P* = 1.08 × 10^−4^) (Figure 3e). Upon continued imbibition (26 HAI), the uORF associated RFs again decreased accompanied by an RF increase in the downstream mORF (Figure 3f). This shows that uORF‐mediated translation regulation during seed germination is highly dynamic. The trade‐off between the RFs at uORFs and mORFs upon germination was also seen in other stages at 26–48 HAI and 48–72 HAI (Figure 3f,g). The genes described are representatives of possible uORF‐mediated translational control during seed germination, of which the molecular mechanism can be further explored.

## DISCUSSION

By deep sequencing ribosome‐protected fragments at five distinct physiological stages of seed germination, we generated a detailed ribosome occupancy profile of this developmental process at single codon resolution. In dry seeds, and at 6 and 26 HAI seeds, 28‐nt sized RFs are most pronounced. In contrast, at later germination stages, the 29‐nt footprints that have been previously reported for Arabidopsis become more prevalent (Bazin et al., 2017; Gelsinger et al., 2020).

The position of ribosome association on an mRNA transcript is pivotal for its translational outcome, since regions such as the 5′ UTR, the CDS and the 3′ UTR have distinct roles in regulating translation. In dry seeds, ribosome association is significantly higher in both the 5′ UTR and 3′ UTR compared with later germination stages, suggesting a higher proportion of untranslated mRNAs. This pattern is consistent with the absence of translation in dry seeds (Figure 1b). Based on previous reports showing that transcripts in dry seeds are predominantly associated with monosomes, we conclude that a ribosome on a given transcript is typically associated with either the 5′ UTR or 3′ UTR, but not both (Bai et al., 2020). Upon imbibition, comparing dry seeds to 6 HAI, the translation of stored mRNAs follows two distinct patterns. Transcripts are either translationally downregulated, showing a high RF signal in the start codon region of dry seeds and an overall reduction in RF at 6 HAI (Figure 2a,b), or upregulated, indicated by the high RF signal in the start codon region in dry seeds and an overall increase in RF at 6 HAI (Figure 2c,d). These two translational trajectories in seeds correspond to two hypotheses regarding the fate of seed‐stored mRNA respectively. (1) *Downregulated upon germination*: This group includes mRNAs that are actively translated during seed maturation, but degraded upon germination. Examples include transcripts encoding LEAs and seed oil body‐associated proteins involved in seed filling. This pattern likely represents the gene expression program during seed maturation, where mRNA translation is gradually attenuated toward the end of development, resulting in the complete loss of translational activity in dry seeds (Bai et al., 2023). (2) *Upregulation upon germination*: These are mRNAs that encode proteins required during early seed germination, such as seed storage proteins. These transcripts accumulate during seed maturation (Baud et al., 2008) and have been associated with the establishment of seed desiccation tolerance (Terrasson et al., 2013). Upon imbibition, they become translationally active which is in agreement with the observation that storage proteins are synthesized during early germination (Galland et al., 2014; Rajjou et al., 2004). The translation of seed‐stored mRNAs at early stages of seed imbibition might have ecological advantages. Early imbibition reflects a period in which seeds can still tolerate desiccation (Maia et al., 2011), therefore, the translation of these seed maturation and stress response proteins could help tolerating recurrent hydration‐dehydration cycles. Moreover, seed storage proteins have been reported as “buffering proteins” for protection against oxidative damage (Nguyen et al., 2015). Their translation might be required for maintaining seed vigor in adverse environments, to increase the chance of seedling survival. This stress surveillance mechanism is specific for seeds before testa rupture and coincides with the stage at which seeds do not tolerate desiccation anymore (Maia et al., 2011). From 26 HAI onwards the transcript abundance and translation of seed storage proteins are sharply reduced (Figure 2c). Moreover, after the initial imbibition there is a general trend of translational downregulation (Figure S2b). This downregulation is mostly the result of a stronger transcriptional than translational upregulation, as indicated by the T+R+ pattern (Figure S2; Table S10). Still this will lead to an increase in protein abundance, however, the increase is less than expected based on the transcript abundance. This phenomenon could be caused by insufficient translational power, since ribosomal abundance only strongly increases from 48 HAI onwards (Bai et al., 2017).

Ribosome stalling during protein translation has significant biological roles, for instance, to provide enough time for molecular processes like nascent peptide folding, and the recruitment of signal recognition particles for ER targeting (Bui & Hoang, 2016; Mercier et al., 2017; Nyathi et al., 2013; Tanner et al., 2009). The amino acids glycine, aspartate, tyrosine, and proline provide the codons for ribosome pausing during seed germination (Figure 1c). This is consistent with previous observations showing that proline, glycine, and aspartate are the most conserved amino acids associated with ribosome stalling in yeast, fruit fly, zebrafish, mouse, and human (Chyzynska et al., 2021). Ribosome stalling also frequently occurs at the aromatic amino acids tyrosine (also identified during seed germination, Figure 1c), phenylalanine, and tryptophan in bacteria (Cymer et al., 2015). Ribosome stalling is described to enhance protein folding and to diminish mRNA decay and protein degradation (Stein & Frydman, 2019). Biological consequences of stalling at specific codons in plants have yet not been described.

This study identified a Kozak‐like consensus sequence that may act as a sequence‐based signal for ribosome pausing at the start codon. An enrichment of A residues at positions −2 and −3 and a G residue at +4 likely contribute to this pausing and influence codon choice, thereby enhancing selectivity of translation (Figure 2d). This Kozak‐like motif‐dependent control is supported by the strong ribosome occupancy at start codons in dry seeds, which decreases upon germination (Figure 1b). Similar mechanisms have been reported in other plant genes, suggesting a broader role in translational regulation (Hang et al., 2024).

lncRNAs have been historically regarded as non‐coding RNA based on their size and coding potential. However, there is increasing evidence that lncRNAs can encode short peptides that play roles in transcriptional regulation, splicing, and protein scaffolding (Bazin et al., 2017; Sebastian‐delaCruz et al., 2021; Wu et al., 2024; Zeng et al., 2018). Moreover, the lncRNA‐encoded small peptide microRPG1 plays a key role in controlling maize seed desiccation by mediating ethylene signaling (Yu et al., 2024). In this study, we have identified over a thousand lncRNAs that are associated with ribosomes (Table S11). The identification of peptides that are encoded by lncRNA ORFs provides strong evidence that these lncRNAs are translated during seed germination (Table S13), which might add an additional layer of gene expression control during seed germination. Not all ribosome‐associated lncRNAs contain an ORF, which could allow them to generate peptides; however, for these lncRNAs, a regulatory role during translation cannot be excluded. Although largely unannotated, lncRNAs have been reported to play a role in regulating seed germination, with examples like *asDOG1* and TE‐lincRNA11195 (Wang et al., 2017; Yatusevich et al., 2017). These unexplored resources of ribosome‐associated lncRNAs open up a plethora of possibilities for understanding translational regulation in seeds.

Translation can also be regulated by uORFs. Well‐studied examples in plants are the CPuORF of the group S basic region leucine zippers (S1 bZIPs), which mediate sucrose‐induced repression of translation (SIRT). SIRT confers the gene expression regulation of the S1 bZIP ATB2/AtbZIP11 upon sucrose treatment (Rook et al., 1998; van der Horst et al., 2020; Wiese et al., 2004). The interaction between uORF‐encoded peptides and the ribosome exit tunnel, where the nascent peptide is released from the ribosome, has been shown to regulate ribosomal arrest, which causes ribosome stalling (Yamashita et al., 2017). In this study, uORFs that regulate gene expression at the translational level were identified. For example, the translation of the uORF of *ABH* (AT5G36210) in dry seeds represses the translation of the *ABH* mORF, whereas at 6 HAI this repression is attenuated (Figure 3e; Table S13). The function of the ABH family protein during germination remains unknown; however, the ABH domain can serve as a core component in the perception of gibberellin, strigolactone, and karrikin, which are known to promote seed germination (Guo et al., 2013; Hernandez‐Garcia et al., 2021; Shimada et al., 2008). This uORF‐mediated gene expression attenuation can also occur upon imbibition, as observed for *DET3* (AT1G12840) where increased ribosome association with the uORF correlates with reduced ribosome association with the mORF from dry to imbibed seeds (Figure 3a,e). This uORF‐mediated repression is specific to 6 HAI; by 26 HAI the ribosome association with the uORF reduces, coinciding with an increase in mORF ribosome association (Figure 3b,c,f). Development‐dependent uORF control of gene expression is seen in all stages during germination, which fine‐tunes gene expression regardless of their transcript level. uORF‐mediated translational control has been described as key in fast adaptive response to environmental stress (Chen & Tarn, 2019). Instant gene expression control during seed imbibition is essential to regulate germination, ensuring it only occurs under conditions that are optimal for seedling establishment.

In conclusion, this study provides the first translational landscape at sub‐codon resolution across seed germination and identified different mechanisms that may regulate translation during seed germination. The RFs in all germination stages showed *bona fide* ribosome association and substantial ribosome dynamics. In dry seeds, we found a relatively high ribosome association with the 5′ and 3′ UTRs, and low association with the CDS, compared with later stages of seed germination where association predominates in the CDS. This finding corresponds to the changes in translational status during seed germination. Translation of lncRNAs is supported by their ribosome association as well as the identification of their encoded peptides. The function of these peptides in seed germination has yet to be elucidated. Finally, previously unannotated uORFs that might adjust translation of the mORF were identified. We propose that the various mechanisms of translational control during seed germination are crucial for enabling rapid responses to environmental changes, thereby ensuring the survival of germinating seeds and contributing to the seed's role in the plant's overall survival strategy.

## MATERIALS AND METHODS

### Plant material

Seeds of the *Arabidopsis thaliana* accession Columbia‐0 (NASC N60000) were used for ribosome profiling. Sampling was performed according to Bai et al. (2017) for three independent biological replicates. Briefly, seeds were germinated in a 22°C incubator under continuous light (143 μmol m^−2^ sec^−1^). Seeds were harvested at 0, 6, 26, 46, and 72 h after imbibition (HAI). The harvested material was frozen in liquid nitrogen followed by freeze‐drying. The dry material was stored at −80°C until further use.

### Ribosome isolation and footprint preparation

The ribosome isolation was performed according to Bai et al. (2017) and the footprint preparation was modified from Hsu et al. (2016). In brief, 50 mg dry seed material was extracted with 2 ml of polysome extraction buffer, PEB (400 mm Tris pH 9.0, 0.25 m sucrose, 200 mm KCl, 35 mm MgCl_2_, 5 mm EGTA, 1 mm phenylmethane sulfonyl fluoride, 5 mm dithiothreitol, 50 μg ml^−1^ cycloheximide, 50 μg ml^−1^ chloramphenicol). A 50‐μl aliquot of supernatant was used to extract total RNA for constructing RNA‐seq libraries (see below). A 200‐μl aliquot of supernatant was used for ribosomal footprints (RF) preparation. This aliquot was treated with RNase I nuclease (Invitrogen/Thermo Fisher Scientific, Breda, the Netherlands; cat. no. AM2294) at 10 U per 10 OD 260 unit ribosome aliquot. Enzyme digestion was performed at 23°C with gentle mixing on a thermal mixer (Eppendorf) for 1.5 h. Nuclease digestion was stopped by adding 15 μl of SUPERase‐in (Thermo Fisher Scientific, Breda, the Netherlands; AM2696). Fragmented RNA was purified and separated by 15% (wt/vol) TBE‐urea PAGE (Thermo Fisher Scientific; EC68852BOX), and gel slices corresponding to fragment sizes between 26 and 34 nt were excised according to the two RNA oligo markers with 26 and 34 nt in size. RFs were recovered from the excised gel slices following the overnight elution method as specified by Ingolia et al. (2012).

### Ribosome and RNA sequencing library construction and sequencing

After obtaining RFs, Ribo‐seq libraries were constructed according to ARTseq/TruSeq Ribo Profile Kit manual and amplified by 13 cycles of PCR with a barcode incorporated in the primer. The PCR products were gel purified using the overnight method described by Ingolia et al. (2012). Equal molarities of the libraries were pooled for single‐end 50‐bp sequencing using an Illumina NovaSeq 6000 sequencer.

For RNA‐seq, a 50‐μl aliquot of supernatant as described above was used to extract total RNA by TriPure Isolation Reagent (Roche, Basel, Switzerland), followed by clean‐up using RNeasy Mini spin columns (Qiagen, Hilden, Germany). The concentration of purified RNA was quantified using nanodrop. Then, 5 μg of total RNA was subjected to rRNA depletion using Illumina Ribo‐Zero Plus rRNA Depletion Kit following the manufacturer's instructions. The rRNA‐depleted RNA was used to construct sequencing libraries using the ARTseq/TruSeq Ribo Profile Kit (Illumina). The circularized cDNA was amplified by 11 cycles of PCR and gel purified using the same procedure for the Ribo‐seq libraries described above. Libraries were barcoded, pooled, and sequenced using an Illumina HiSeq 2500 machine. The sequencing data were submitted to sequence read archive (SRA) from NCBI with BioProject ID PRJNA1169673.

### Adaptor processing, quality assessment, and alignment

FASTQ files were obtained, and all data analysis steps were performed in house, using a combination of command line software tools and python scripts. Adaptors were trimmed off with the cutadapt program (Martin, 2011) using the following adaptor sequence: CTGTAGGCACCATCAAT, reads smaller than 18 bp were removed; Quality reports of raw and trimmed read sets were generated with the FastQC program. The getfasta command from the bedtools program (Quinlan & Hall, 2010) was used to extract strand‐specific sequences in FASTA format for the annotated rRNA, tRNA, miRNA, snRNA, snoRNA, and lncRNA sequences in the Arabidopsis genome using the Araport11 genome annotation (Cheng et al., 2017). The trimmed and quality‐filtered reads were mapped to these RNA sequences with the short sequence sensitive mapping tool Bowtie v1.2.3, following the strategy of mapping, filtering unmapped reads and mapping to another reference in the reference order of rRNA, tRNA, snRNA, snoRNA, miRNA, lncRNA, mRNA, and genome (Langmead et al., 2009). Each read was only allowed to map once to the best location with at most one mismatch and only to the coding strand except in the final step when mapping to the genome. The Arabidopsis genome sequence was retrieved from The Arabidopsis Information Resource (https://www.arabidopsis.org), version 10 and the Araport11 genome annotation was downloaded from Ensembl Plants (https://plants.ensembl.org/index.html).

### Meta‐gene analysis, data visualization

To verify and select specific reads showing periodicity of ribosome association, the R package RiboWaltz was used (Lauria et al., 2018). Transcriptome alignment (binary alignment map, BAM) files and Araport11 annotation gff file were provided as input (Cheng et al., 2017). For data visualization, the corresponding FASTA sequences (e.g., lncRNA, transcriptome, genome) generated for Bowtie mapping were used as reference. BAM files were filtered for specific ribosome‐protected read length and visualized using the IGV genome browser (Robinson et al., 2011).

### Differential translation comparison, clustering, and gene ontology term enrichment analysis

Raw read counts were normalized with the DESeq2 R package and PCA analysis was performed using the plotPCA function from DESeq2 based on normalized read counts. Differential translation analysis was performed by counting reads in the transcriptome alignments using the featureCounts function from the Subread v 2.0.1 R package with gff files as reference (Liao et al., 2013). DESeq2 was applied to perform differential translation analysis between five time points. Statistical analysis was performed using the design formula ~ assay + condition + assay:condition. Using the likelihood ratio test of DESeq2, which removes the interaction term in the reduced model, the difference in TE by comparing the ribosome‐associated RNA to total RNA between two conditions can be tested. The length normalized counts in rpkm for each gene were calculated based on the formula rpkm = coding sequence (CDS) mapped reads/(CDS length/1000 × total reads/1 000 000) by using normalized read counts. Genes with an 5% FDR‐adjusted *P*‐value <0.05 and at least of twofold change between ribosome‐associated RNA and total RNA were considered as translationally regulated. Read counts of various feature types of interest (e.g., CDSs, UTRs) were calculated separately using the featureCounts function from Subread v 2.0.1 with gff files as reference. For gene ontology (GO) enrichment the Fisher's exact test was performed using the SciPy Python library (version 1.5.2) and a 5% FDR was applied for multiple testing using the multitest.multipletests function with method Benjamini/Hochberg from the Python statsmodels module (version 0.12.1). GO annotation was performed using the GO_slim file from the TAIR website (https://www.arabidopsis.org/). The start codon‐enriched genes in dry seeds were identified by calculating the proportion of P sites at every position across the entire CDS. Genes with more than 10% of total P sites at the start codon were selected, and those with start codon P site counts greater than 30 were further retained as startcodon‐enriched genes The PANTHER server (https://pantherdb.org) was used for GO enrichment analysis (release 20221013) (Thomas et al., 2022).

### Long non‐coding RNA analysis

Long non‐coding RNA in both total RNA (total lncRNA) and ribosome‐associated RNA (ribo lncRNA) is classified with the Araport11_gene_type file (2019 update) from the Araport11 genome release. lncRNA with rpkm >5 in all three biological replicates were kept for both total lncRNA and ribo lncRNA. TE of lncRNA was calculated in the same way as for mRNA, as the ratio of ribo lncRNA to total lncRNA. lncRNA that were detected both in RNA‐seq and Ribo‐seq and lncRNAs with detected ORFs with predicted ORFs by systemPipeR that were significantly changed in TE were considered “translationally regulated.”

### Mass‐spectra based proteomic analysis of lncRNA‐encoded peptides during seed germination

The peptide sequence encoded by the short open reading frame (sORF) of the identified lncRNAs was predicted by systemPipeR's predORF function (Backman & Girke, 2016) and stored in FASTA format as input for proteomic analyses. The proteomic data were retrieved from Bai et al. (2021) at ProteomeXchange with accession PXD027345 for mass spectrometry based proteomic analysis during seed germination. Identification of proteins from the raw MS Data was done using Proteome Discoverer (PD) 2.4 SP1 (Thermo Fisher Scientific, USA) with default settings. PD was set up to search the protein database of *Arabidopsis thaliana*, downloaded from TAIR (https://www.arabidopsis.org/, version TAIR10_pep_20101214). Human keratin and protease mixture sequences were used as the contaminant database for proteomic searching. A FDR cutoff of 1% was applied for the peptide and protein search. The processed data output was then exported to Excel for further usage. The peptides identified from all biological replicates in each stage were selected as the identified peptides encoded by the identified lncRNAs.

### uORF analysis

SystemPipeR's predORF function was used for uORF prediction by searching potential ORFs in the 5′ UTR region of each gene transcript. ORFs longer than 60 bp (20 aa) were kept and converted into uORF annotation in gff format. 5′ UTR sequences were extracted based on the Araport11 genome and annotation. Ribosome‐associated uORFs and ribosome‐associated CDSs reads were extracted from Ribo‐seq data based on each annotation respectively. Genes with both ribosome‐associated uORFs and CDSs with rpkm >5 in all three biological replicates were kept for differential expression (ribosome abundance) analysis. Ribosome‐associated uORFs and ribosome‐associated CDSs were calculated separately and compared with each other. Comparisons between CDS and their associated uORFs were calculated using the generalized linear model of DESeq2 as described above, using read counts from Ribo‐seq of CDS and uORF as input to DESeq2. Genes with significant changes in RF count in uORFs and CDSs were identified using DESeq2, with adjusted *p*‐values calculated using the Benjamini–Hochberg method (*P*adj <0.05). Translationally regulated genes were defined as those showing opposite regulation patterns in log_2_Fold change in RF count of uORF and CDS between consecutive time points during seed germination.

## AUTHOR CONTRIBUTIONS

BB and LB designed the experiments. BB performed the experiments. BB, HN and LB directed the design of analysis approaches. BB, RQ and WS conducted the analyses. BB and LB drafted the manuscript. All authors participated in revising and editing the manuscript.

## CONFLICT OF INTEREST

Authors declare no competing interests.

## Supporting information


**Figure S1.** Meta‐gene heatmap displaying the signal associated with the 5′ end (upper panel) and 3′ end (lower panel) of the ribosome footprints around the start and the stop codon for fragment lengths 27, 28, 29 nt following dry, 6, 26, 48, and 72 h after imbibition (HAI) for all three biological replicates.
**Figure S2.** Translational regulated genes during seed germination.
**Figure S3.** Comparison between results obtained using ribosome sequencing in this study and the polysome profiling analyses during seed germination (Bai et al., 2017) at the (a) transcriptome level, (b) translatome level, and (c) translation efficiency level.
**Figure S4.** Translationally regulated lncRNA during seed germination.
**Figure S5.** Investigation of the ribosome association of the 176 dry seeds start codon‐enriched genes during seed maturation and seed germination.

**Table S1.** Mapping percentage of different RNAs.
**Table S2.** Mapping percentage of the ribosome reads at different mRNA regions in all samples.
**Table S3.** Mapping percentage of the ribosome reads at start and stop codon in all samples.
**Table S4.** Codon usage index during seed germination. The value is normalize by the corresponding codon frequencies in coding sequences.
**Table S5.** Description of 176 start codon‐enriched genes in dry seed.
**Table S6.** GO term of 176 start codon‐enriched genes in dry seed.
**Table S7.** Chi‐square test for each nucleotide position around the AUG start codon for the codon enriched‐genes in dry seeds. The asterisk sign indicates the significant different in the frequency in that nt position.
**Table S8.** GO enrichment analysis of the translationally regulated genes across seed germination.
**Table S9.** Clusters of mRNA changed in translational efficiency during seed germination.
**Table S10.** GO enrichment analysis of the translationally regulated genes by clusters across seed germination.
**Table S11.** Detected non‐coding RNA during seed germination.
**Table S12.** Translationally regulated long non‐coding RNA (lncRNA) during seed germination.
**Table S13.** Total proteomic identification of lncRNA‐encoded proteins at different stages during seed germination.
**Table S14.** Genes that are translationally regulated by uORF during seed germination.

## Data Availability

The sequencing data generated in this study have been deposited in the NCBI Sequence Read Archive (SRA) under accession code PRJNA1169673.

## References

[tpj70663-bib-0001] Backman, T.W.H.B. & Girke, T. (2016) systemPipeR: NGS workflow and report generation environment. BMC Bioinformatics, 17, 388. 27650223 10.1186/s12859-016-1241-0 PMC5029110

[tpj70663-bib-0002] Bai, B. , Peviani, A. , van der Horst, S. , Gamm, M. , Snel, B. , Bentsink, L. et al. (2017) Extensive translational regulation during seed germination revealed by polysomal profiling. New Phytologist, 214, 233–244. 27935038 10.1111/nph.14355 PMC5347915

[tpj70663-bib-0003] Bai, B. , Schiffthaler, B. , van der Horst, S. , Willems, L. , Vergara, A. , Karlstrom, J. et al. (2023) SeedTransNet: a directional translational network revealing regulatory patterns during seed maturation and germination. Journal of Experimental Botany, 74, 2416–2432. 36208446 10.1093/jxb/erac394 PMC10082931

[tpj70663-bib-0004] Bai, B. , van der Horst, N. , Cordewener, J.H. , America, A.H.P. , Nijveen, H. & Bentsink, L. (2021) Delayed protein changes during seed germination. Frontiers in Plant Science, 12, 735719. 34603360 10.3389/fpls.2021.735719 PMC8480309

[tpj70663-bib-0005] Bai, B. , van der Horst, S. , Cordewener, J.H.G. , America, T. , Hanson, J. & Bentsink, L. (2020) Seed‐stored mRNAs that are specifically associated to monosomes are translationally regulated during germination. Plant Physiology, 182, 378–392. 31527088 10.1104/pp.19.00644 PMC6945870

[tpj70663-bib-0006] Baud, S. , Dubreucq, B. , Miquel, M. , Rochat, C. & Lepiniec, L. (2008) Storage reserve accumulation in Arabidopsis: metabolic and developmental control of seed filling. The Arabidopsis Book, 6, e0113. 22303238 10.1199/tab.0113 PMC3243342

[tpj70663-bib-0007] Bazin, J. , Baerenfaller, K. , Gosai, S.J. , Gregory, B.D. , Crespi, M. & Bailey‐Serres, J. (2017) Global analysis of ribosome‐associated noncoding RNAs unveils new modes of translational regulation. Proceedings of the National Academy of Sciences of the United States of America, 114, E10018–E10027. 29087317 10.1073/pnas.1708433114 PMC5699049

[tpj70663-bib-0008] Brown, A. , Shao, S. , Murray, J. , Hegde, R.S. & Ramakrishnan, V. (2015) Structural basis for stop codon recognition in eukaryotes. Nature, 524, 493–496. 26245381 10.1038/nature14896 PMC4591471

[tpj70663-bib-0009] Bui, P.T. & Hoang, T.X. (2016) Folding and escape of nascent proteins at ribosomal exit tunnel. Journal of Chemical Physics, 144, 095102. 26957181 10.1063/1.4943042

[tpj70663-bib-0010] Chen, H.H. & Tarn, W.Y. (2019) uORF‐mediated translational control: recently elucidated mechanisms and implications in cancer. RNA Biology, 16, 1327–1338. 31234713 10.1080/15476286.2019.1632634 PMC6779392

[tpj70663-bib-0011] Cheng, C.Y. , Krishnakumar, V. , Chan, A.P. , Thibaud‐Nissen, F. , Schobel, S. & Town, C.D. (2017) Araport11: a complete reannotation of the *Arabidopsis thaliana* reference genome. The Plant Journal: For Cell and Molecular Biology, 89, 789–804. 27862469 10.1111/tpj.13415

[tpj70663-bib-0012] Chyzynska, K. , Labun, K. , Jones, C. , Grellscheid, S.N. & Valen, E. (2021) Deep conservation of ribosome stall sites across RNA processing genes. NAR Genomics and Bioinformatics, 3, lqab038. 34056595 10.1093/nargab/lqab038 PMC8152447

[tpj70663-bib-0013] Cymer, F. , Hedman, R. , Ismail, N. & von Heijne, G. (2015) Exploration of the arrest peptide sequence space reveals arrest‐enhanced variants. The Journal of Biological Chemistry, 290, 10208–10215. 25713070 10.1074/jbc.M115.641555 PMC4400336

[tpj70663-bib-0014] Dure, L. & Waters, L. (1965) Long‐lived messenger RNA: evidence from cotton seed germination. Science, 147, 410–412. 14221492 10.1126/science.147.3656.410

[tpj70663-bib-0015] Galland, M. , Huguet, R. , Arc, E. , Cueff, G. , Job, D. & Rajjou, L. (2014) Dynamic proteomics emphasizes the importance of selective mRNA translation and protein turnover during Arabidopsis seed germination. Molecular & Cellular Proteomics: MCP, 13, 252–268. 24198433 10.1074/mcp.M113.032227 PMC3879618

[tpj70663-bib-0016] Gelsinger, D.R. , Dallon, E. , Reddy, R. , Mohammad, F. , Buskirk, A.R. & DiRuggiero, J. (2020) Ribosome profiling in archaea reveals leaderless translation, novel translational initiation sites, and ribosome pausing at single codon resolution. Nucleic Acids Research, 48, 5201–5216. 32382758 10.1093/nar/gkaa304 PMC7261190

[tpj70663-bib-0017] Guo, Y. , Chen, Y. , Wang, Y. , Wu, X. , Zhang, X. , Mao, W. et al. (2023) The translational landscape of bread wheat during grain development. The Plant Cell, 35, 1848–1867. 36905284 10.1093/plcell/koad075 PMC10226598

[tpj70663-bib-0018] Guo, Y. , Zheng, Z. , La Clair, J.J. , Chory, J. & Noel, J.P. (2013) Smoke‐derived karrikin perception by the alpha/beta‐hydrolase KAI2 from Arabidopsis. Proceedings of the National Academy of Sciences of the United States of America, 110, 8284–8289. 23613584 10.1073/pnas.1306265110 PMC3657771

[tpj70663-bib-0019] Han, Y. , Gao, X. , Liu, B. , Wan, J. , Zhang, X. & Qian, S.B. (2014) Ribosome profiling reveals sequence‐independent post‐initiation pausing as a signature of translation. Cell Research, 24, 842–851. 24903108 10.1038/cr.2014.74 PMC4085768

[tpj70663-bib-0020] Hang, R. , Li, H. , Liu, W. , Wang, R. , Hu, H. , Chen, M. et al. (2024) HOT3/eIF5B1 confers Kozak motif‐dependent translational control of photosynthesis‐associated nuclear genes for chloroplast biogenesis. Nature Communications, 15, 9878. 10.1038/s41467-024-54194-1 PMC11564774 39543117

[tpj70663-bib-0021] Hernandez‐Garcia, J. , Briones‐Moreno, A. & Blazquez, M.A. (2021) Origin and evolution of gibberellin signaling and metabolism in plants. Seminars in Cell & Developmental Biology, 109, 46–54. 32414681 10.1016/j.semcdb.2020.04.009

[tpj70663-bib-0022] Heyer, E.E. & Moore, M.J. (2016) Redefining the translational status of 80S monosomes. Cell, 164, 757–769. 26871635 10.1016/j.cell.2016.01.003

[tpj70663-bib-0023] Hsu, P.Y. , Calviello, L. , Wu, H.Y.L. , Li, F.W. , Rothfels, C.J. , Ohler, U. et al. (2016) Super‐resolution ribosome profiling reveals unannotated translation events in Arabidopsis. Proceedings of the National Academy of Sciences of the United States of America, 113, E7126–E7135. 27791167 10.1073/pnas.1614788113 PMC5111709

[tpj70663-bib-0024] Ingolia, N.T. , Brar, G.A. , Rouskin, S. , McGeachy, A.M. & Weissman, J.S. (2012) The ribosome profiling strategy for monitoring translation in vivo by deep sequencing of ribosome‐protected mRNA fragments. Nature Protocols, 7, 1534–1550. 22836135 10.1038/nprot.2012.086 PMC3535016

[tpj70663-bib-0025] Ingolia, N.T. , Ghaemmaghami, S. , Newman, J.R. & Weissman, J.S. (2009) Genome‐wide analysis in vivo of translation with nucleotide resolution using ribosome profiling. Science, 324, 218–223. 19213877 10.1126/science.1168978 PMC2746483

[tpj70663-bib-0026] Joshi, C.P. , Zhou, H. , Huang, X. & Chiang, V.L. (1997) Context sequences of translation initiation codon in plants. Plant Molecular Biology, 35, 993–1001. 9426620 10.1023/a:1005816823636

[tpj70663-bib-0027] Juntawong, P. , Girke, T. , Bazin, J. & Bailey‐Serres, J. (2014) Translational dynamics revealed by genome‐wide profiling of ribosome footprints in Arabidopsis. Proceedings of the National Academy of Sciences of the United States of America, 111, E203–E212. 24367078 10.1073/pnas.1317811111 PMC3890782

[tpj70663-bib-0028] Langmead, B. , Trapnell, C. , Pop, M. & Salzberg, S.L. (2009) Ultrafast and memory‐efficient alignment of short DNA sequences to the human genome. Genome Biology, 10, R25. 19261174 10.1186/gb-2009-10-3-r25 PMC2690996

[tpj70663-bib-0029] Lauria, F. , Tebaldi, T. , Bernabo, P. , Groen, E.J.N. , Gillingwater, T.H. & Viero, G. (2018) riboWaltz: optimization of ribosome P‐site positioning in ribosome profiling data. PLoS Computational Biology, 14, e1006169. 30102689 10.1371/journal.pcbi.1006169 PMC6112680

[tpj70663-bib-0030] Liao, Y. , Smyth, G.K. & Shi, W. (2013) The subread aligner: fast, accurate and scalable read mapping by seed‐and‐vote. Nucleic Acids Research, 41, e108. 23558742 10.1093/nar/gkt214 PMC3664803

[tpj70663-bib-0031] Liu, M.J. , Wu, S.H. & Chen, H.M. (2012) Widespread translational control contributes to the regulation of Arabidopsis photomorphogenesis. Molecular Systems Biology, 8, 566. 22252389 10.1038/msb.2011.97 PMC3296358

[tpj70663-bib-0032] Maia, J. , Dekkers, B.J. , Provart, N.J. , Ligterink, W. & Hilhorst, H.W. (2011) The re‐establishment of desiccation tolerance in germinated *Arabidopsis thaliana* seeds and its associated transcriptome. PLoS One, 6, e29123. 22195004 10.1371/journal.pone.0029123 PMC3237594

[tpj70663-bib-0033] Martin, M. (2011) Cutadapt removes adapter sequences from high‐throughput sequencing reads. EMBnet.journal, 17, 10–12.

[tpj70663-bib-0034] Meijer, H.A. & Thomas, A.A. (2003) Ribosomes stalling on uORF1 in the *Xenopus* Cx41 5′ UTR inhibit downstream translation initiation. Nucleic Acids Research, 31, 3174–3184. 12799445 10.1093/nar/gkg429 PMC162333

[tpj70663-bib-0035] Mercier, E. , Holtkamp, W. , Rodnina, M.V. & Wintermeyer, W. (2017) Signal recognition particle binds to translating ribosomes before emergence of a signal anchor sequence. Nucleic Acids Research, 45, 11858–11866. 29149347 10.1093/nar/gkx888 PMC5714171

[tpj70663-bib-0036] Nguyen, T.P. , Cueff, G. , Hegedus, D.D. , Rajjou, L. & Bentsink, L. (2015) A role for seed storage proteins in Arabidopsis seed longevity. Journal of Experimental Botany, 66, 6399–6413. 26184996 10.1093/jxb/erv348 PMC4588887

[tpj70663-bib-0037] Nyathi, Y. , Wilkinson, B.M. & Pool, M.R. (2013) Co‐translational targeting and translocation of proteins to the endoplasmic reticulum. Biochimica et Biophysica Acta, 1833, 2392–2402. 23481039 10.1016/j.bbamcr.2013.02.021

[tpj70663-bib-0038] Quinlan, A.R. & Hall, I.M. (2010) BEDTools: a flexible suite of utilities for comparing genomic features. Bioinformatics, 26, 841–842. 20110278 10.1093/bioinformatics/btq033 PMC2832824

[tpj70663-bib-0039] Rajjou, L. , Gallardo, K. , Debeaujon, I. , Vandekerckhove, J. , Job, C. & Job, D. (2004) The effect of alpha‐amanitin on the Arabidopsis seed proteome highlights the distinct roles of stored and neosynthesized mRNAs during germination. Plant Physiology, 134, 1598–1613. 15047896 10.1104/pp.103.036293 PMC419834

[tpj70663-bib-0040] Robinson, J.T. , Thorvaldsdottir, H. , Winckler, W. , Guttman, M. , Lander, E.S. , Getz, G. et al. (2011) Integrative genomics viewer. Nature Biotechnology, 29, 24–26. 10.1038/nbt.1754 PMC3346182 21221095

[tpj70663-bib-0041] Rook, F. , Gerrits, N. , Kortstee, A. , van Kampen, M. , Borrias, M. , Weisbeek, P. et al. (1998) Sucrose‐specific signalling represses translation of the Arabidopsis ATB2 bZIP transcription factor gene. The Plant Journal: For Cell and Molecular Biology, 15, 253–263. 9721683 10.1046/j.1365-313x.1998.00205.x

[tpj70663-bib-0042] Sano, N. , Rajjou, L. , North, H.M. , Debeaujon, I. , Marion‐Poll, A. & Seo, M. (2016) Staying alive: molecular aspects of seed longevity. Plant & Cell Physiology, 57, 660–674. 26637538 10.1093/pcp/pcv186

[tpj70663-bib-0043] Sebastian‐delaCruz, M. , Gonzalez‐Moro, I. , Olazagoitia‐Garmendia, A. , Castellanos‐Rubio, A. & Santin, I. (2021) The role of lncRNAs in gene expression regulation through mRNA stabilization. Noncoding RNA, 7, 3. 33466464 10.3390/ncrna7010003 PMC7839045

[tpj70663-bib-0044] Shimada, A. , Ueguchi‐Tanaka, M. , Nakatsu, T. , Nakajima, M. , Naoe, Y. , Ohmiya, H. et al. (2008) Structural basis for gibberellin recognition by its receptor GID1. Nature, 456, 520–523. 19037316 10.1038/nature07546

[tpj70663-bib-0045] Stein, K.C. & Frydman, J. (2019) The stop‐and‐go traffic regulating protein biogenesis: how translation kinetics controls proteostasis. Journal of Biological Chemistry, 294, 2076–2084. 30504455 10.1074/jbc.REV118.002814 PMC6369277

[tpj70663-bib-0046] Tanner, D.R. , Cariello, D.A. , Woolstenhulme, C.J. , Broadbent, M.A. & Buskirk, A.R. (2009) Genetic identification of nascent peptides that induce ribosome stalling. The Journal of Biological Chemistry, 284, 34809–34818. 19840930 10.1074/jbc.M109.039040 PMC2787343

[tpj70663-bib-0047] Terrasson, E. , Buitink, J. , Righetti, K. , Ly Vu, B. , Pelletier, S. , Zinsmeister, J. et al. (2013) An emerging picture of the seed desiccome: confirmed regulators and newcomers identified using transcriptome comparison. Frontiers in Plant Science, 4, 497. 24376450 10.3389/fpls.2013.00497 PMC3859232

[tpj70663-bib-0048] Thomas, P.D. , Ebert, D. , Muruganujan, A. , Mushayahama, T. , Albou, L.P. & Mi, H. (2022) PANTHER: making genome‐scale phylogenetics accessible to all. Protein Science, 31, 8–22. 34717010 10.1002/pro.4218 PMC8740835

[tpj70663-bib-0049] Ulitsky, I. & Bartel, D.P. (2013) lincRNAs: genomics, evolution, and mechanisms. Cell, 154, 26–46. 23827673 10.1016/j.cell.2013.06.020 PMC3924787

[tpj70663-bib-0050] van der Horst, S. , Filipovska, T. , Hanson, J. & Smeekens, S. (2020) Metabolite control of translation by conserved peptide uORFs: the ribosome as a metabolite multisensor. Plant Physiology, 182, 110–122. 31451550 10.1104/pp.19.00940 PMC6945846

[tpj70663-bib-0051] Wang, A. , Hu, J. , Gao, C. , Chen, G. , Wang, B. , Lin, C. et al. (2019) Genome‐wide analysis of long non‐coding RNAs unveils the regulatory roles in the heat tolerance of Chinese cabbage (*Brassica rapa* ssp. *chinensis*). Scientific Reports, 9, 5002. 30899041 10.1038/s41598-019-41428-2 PMC6428831

[tpj70663-bib-0052] Wang, D. , Qu, Z. , Yang, L. , Zhang, Q. , Liu, Z.H. , Do, T. et al. (2017) Transposable elements (TEs) contribute to stress‐related long intergenic noncoding RNAs in plants. The Plant Journal: For Cell and Molecular Biology, 90, 133–146. 28106309 10.1111/tpj.13481 PMC5514416

[tpj70663-bib-0053] Waters, L.C. & Dure, L.S. (1966) Ribonucleic acid synthesis in germinating cotton seeds. Journal of Molecular Biology, 19, 1–27. 5967284 10.1016/s0022-2836(66)80046-3

[tpj70663-bib-0054] Wiese, A. , Elzinga, N. , Wobbes, B. & Smeekens, S. (2004) A conserved upstream open reading frame mediates sucrose‐induced repression of translation. The Plant Cell, 16, 1717–1729. 15208401 10.1105/tpc.019349 PMC514156

[tpj70663-bib-0055] Wu, H. , Yang, L. & Chen, L.L. (2017) The diversity of long noncoding RNAs and their generation. Trends in Genetics: TIG, 33, 540–552. 28629949 10.1016/j.tig.2017.05.004

[tpj70663-bib-0056] Wu, H.L. , Ai, Q. , Teixeira, R.T. , Nguyen, P.H.T. , Song, G. , Montes, C. et al. (2024) Improved super‐resolution ribosome profiling reveals prevalent translation of upstream ORFs and small ORFs in Arabidopsis. The Plant Cell, 36, 510–539. 38000896 10.1093/plcell/koad290 PMC10896292

[tpj70663-bib-0057] Yamashita, Y. , Takamatsu, S. , Glasbrenner, M. , Becker, T. , Naito, S. & Beckmann, R. (2017) Sucrose sensing through nascent peptide‐meditated ribosome stalling at the stop codon of Arabidopsis bZIP11 uORF2. FEBS Letters, 591, 1266–1277. 28369795 10.1002/1873-3468.12634

[tpj70663-bib-0058] Yashina, S. , Gubin, S. , Maksimovich, S. , Yashina, A. , Gakhova, E. & Gilichinsky, D. (2012) Regeneration of whole fertile plants from 30,000‐y‐old fruit tissue buried in Siberian permafrost. Proceedings of the National Academy of Sciences of the United States of America, 109, 4008–4013. 22355102 10.1073/pnas.1118386109 PMC3309767

[tpj70663-bib-0059] Yatusevich, R. , Fedak, H. , Ciesielski, A. , Krzyczmonik, K. , Kulik, A. , Dobrowolska, G. et al. (2017) Antisense transcription represses Arabidopsis seed dormancy QTL DOG1 to regulate drought tolerance. EMBO Reports, 18, 2186–2196. 29030481 10.15252/embr.201744862 PMC5709759

[tpj70663-bib-0060] Yu, Y. , Li, W. , Liu, Y. , Liu, Y. , Zhang, Q. , Ouyang, Y. et al. (2024) A *Zea* genus‐specific micropeptide controls kernel dehydration in maize. Cell, 188, 44–59.e21. 39536747 10.1016/j.cell.2024.10.030

[tpj70663-bib-0061] Yu, Y. , Zhang, Y. , Chen, X. & Chen, Y. (2019) Plant noncoding RNAs: hidden players in development and stress responses. Annual Review of Cell and Developmental Biology, 35, 407–431. 10.1146/annurev-cellbio-100818-125218 PMC8034839 31403819

[tpj70663-bib-0062] Zeng, C. , Fukunaga, T. & Hamada, M. (2018) Identification and analysis of ribosome‐associated lncRNAs using ribosome profiling data. BMC Genomics, 19, 414. 29843593 10.1186/s12864-018-4765-z PMC5975437

[tpj70663-bib-0063] Zhang, Y. , Xiao, Z. , Zou, Q. , Fang, J. , Wang, Q. , Yang, X. et al. (2017) Ribosome profiling reveals genome‐wide cellular translational regulation upon heat stress in *Escherichia coli* . Genomics, Proteomics & Bioinformatics, 15, 324–330. 10.1016/j.gpb.2017.04.005 PMC5673677 29031842

[tpj70663-bib-0064] Zhu, X.T. , Zhou, R. , Che, J. , Zheng, Y.Y. , Tahir Ul Qamar, M. , Feng, J.W. et al. (2023) Ribosome profiling reveals the translational landscape and allele‐specific translational efficiency in rice. Plant Communications, 4, 100457. 36199246 10.1016/j.xplc.2022.100457 PMC10030323

