# Supplementary material for: Translational landscape during seed germination revealed by ribosome profiling
Source: Plant J. 2026 Jan 7;125(1):e70663. doi: 10.1111/tpj.70663 (PMC12778895; doi:10.1111/tpj.70663)
Supplement: Supplementary file 1 — Figure S1. Meta‐gene heatmap displaying the signal associated with the 5′ end (upper panel) and 3′ end (lower panel) of the ribosome footprints around the start and the stop codon for fragment lengths 27, 28, 29 nt following dry, 6, 26, 48, and 72 h after imbibition (HAI) for all three biological replicates. Figure S2. Translational regulated genes during seed germination. Figure S3. Comparison between results obtained using ribosome sequencing in this study and the polysome profiling analyses during seed germination (Bai et al., 2017) at the (a) transcriptome level, (b) translatome level, and (c) translation efficiency level. Figure S4. Translationally regulated lncRNA during seed germination. Figure S5. Investigation of the ribosome association of the 176 dry seeds start codon‐enriched genes during seed maturation and seed germination. [file TPJ-125-0-s001.pdf]

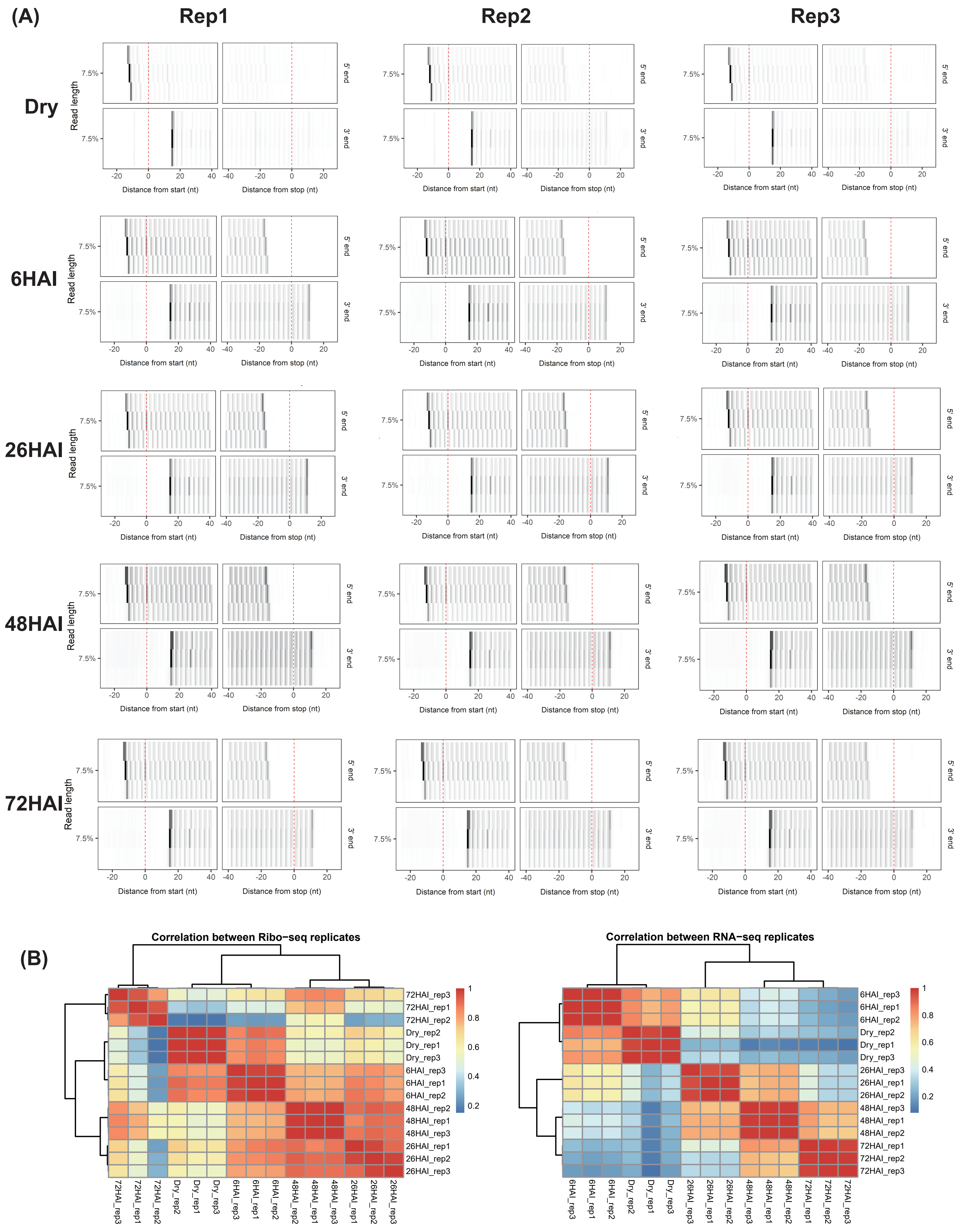

Figure S1. Data reproducibiliy. (A) Meta-gene heatmap displaying the signal associated with the 5' end (upper panel) and 3' end (lower panel) of the ribosome footprints around the start and the stop codon for fragment lengths 27, 28, 29 nt following dry, 6, 26, 48 and 72 hours after imbibition (HAI) with all three biological replicates. (B) The heatmap view of correlation for Ribo-seq and RNA-seq biological replicates.

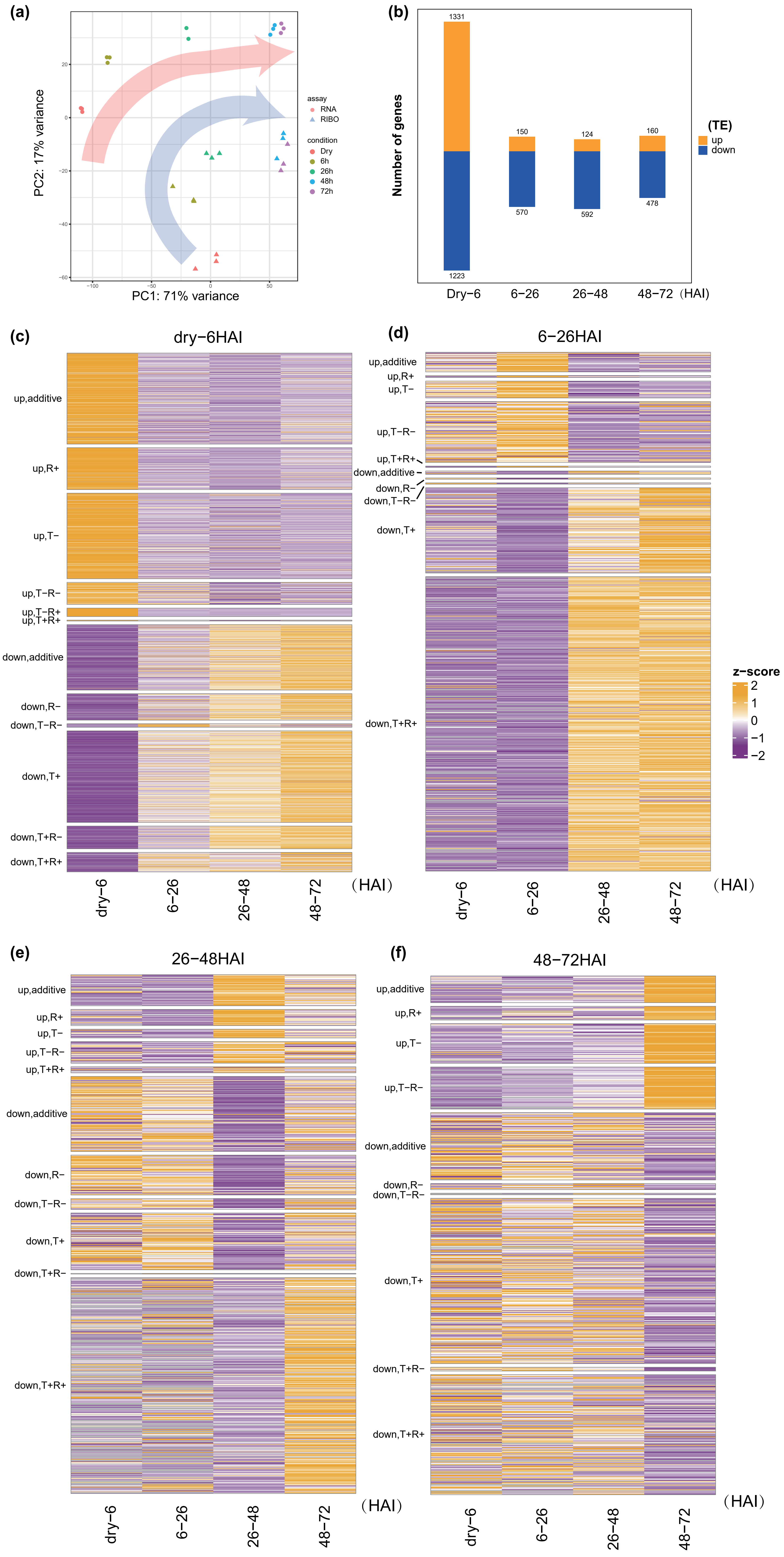

Figure S2. Translational regulated genes during seed germination. (a) PCA plot of total RNA-seq and Ribo-seq data during seed germination. (b) Number of genes with a changed translational efficiency (TE) for different transitions during seed germination. (c-f) Translational clusters for genes with changed TE at dry to 6 HAI (c), 6 to 26 HAI (d), 26 to 48 HAI (e) and 48 to 72 HAI (f) respectively and their translation dynamics during seed germination. Translational clusters are defined by the changes of total RNA (T) and ribosome associated RNA (R) that lead to the changed TE. “+” and “-“ signs indicate the up-/down- regulated at each level. “additive” indicates the significant TE changes are contributed by both T and R level changes. The values represent the fold change of the respective comparison followed by z-score transformation across time points. The yellow and purple represent the up and down of TE changes in the respective comparison.

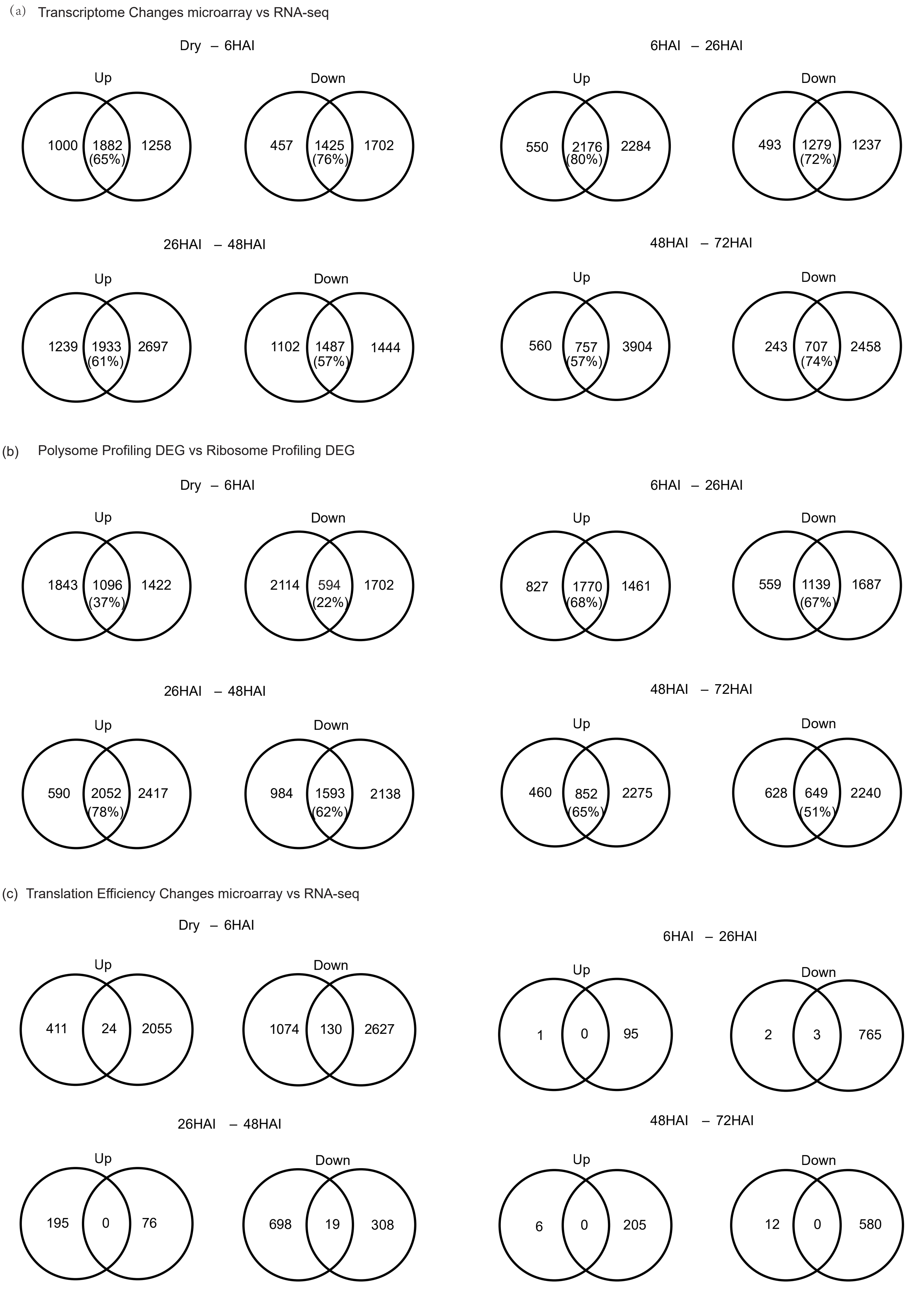

Figure S3. Comparison between results obtained using ribosome sequencing in this study and the polysome profiling analyses during seed germination (Bai et al., 2017) at the (a) transcriptome level, (b) translatome level and (c) translation efficiency level.

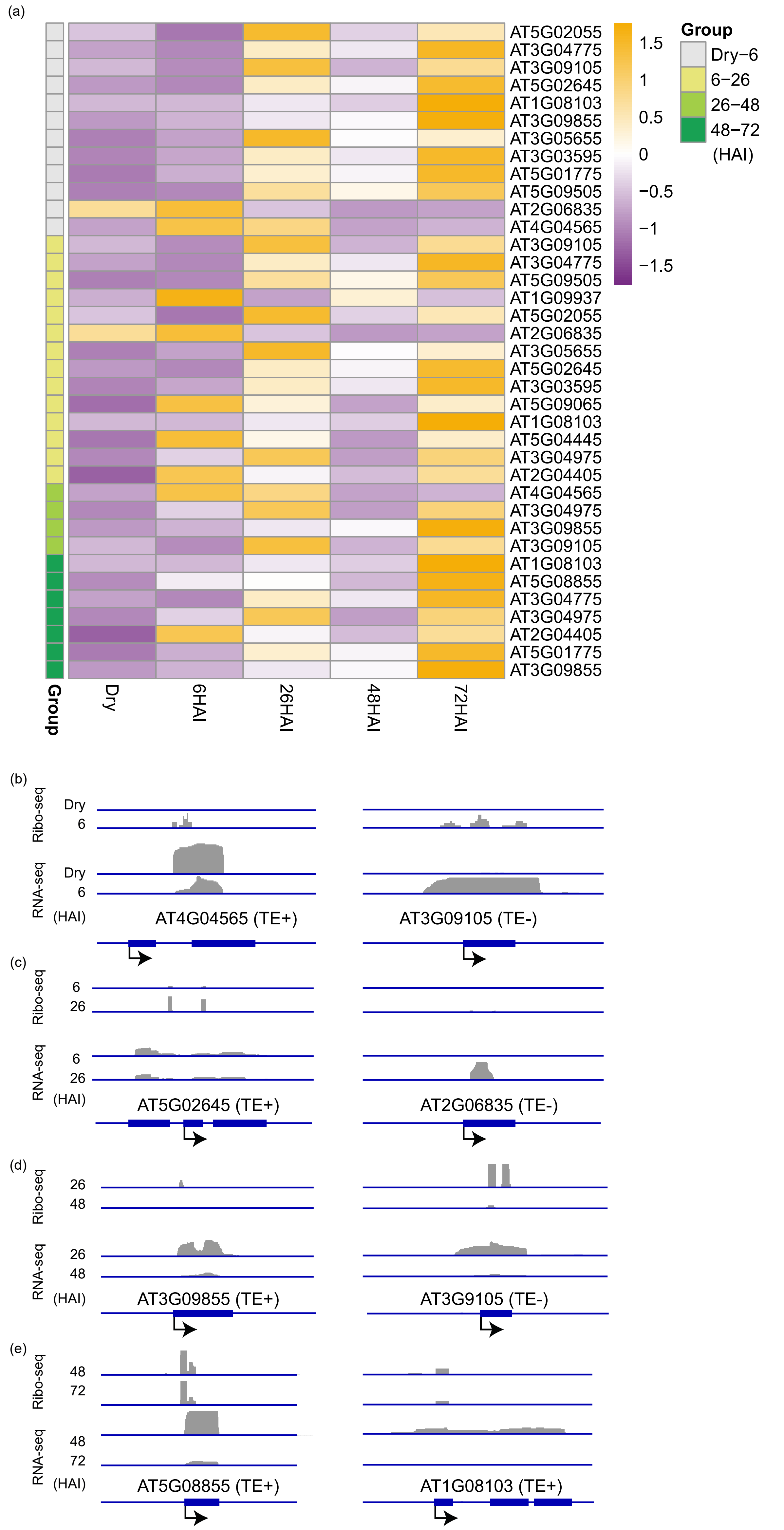

Figure S4. Translationally regulated lncRNA during seed germination. (a) lncRNAs with change in translational efficiency (TE) at each germination stage. The heatmap displays z-scores of TE per lncRNA at each stage, higher values indicate above-average TE, and the Groups represent lncRNAs that change in TE at the stages where the TE changes. (b-e) Coverage plots showing translation (Ribo-seq) and transcription (RNA-seq) read densities during seed germination for representative lncRNAs. (b) dry to 6HAI. (c) 6 to 26 HAI. (d) 26 to 48 HAI. (e) 48 to 72 HAI. TE+ and TE- represent the up-/down-regulation of TE changes. Data from three independent biological replicates were pooled, and results are presented using the same read density scale for the compared groups.

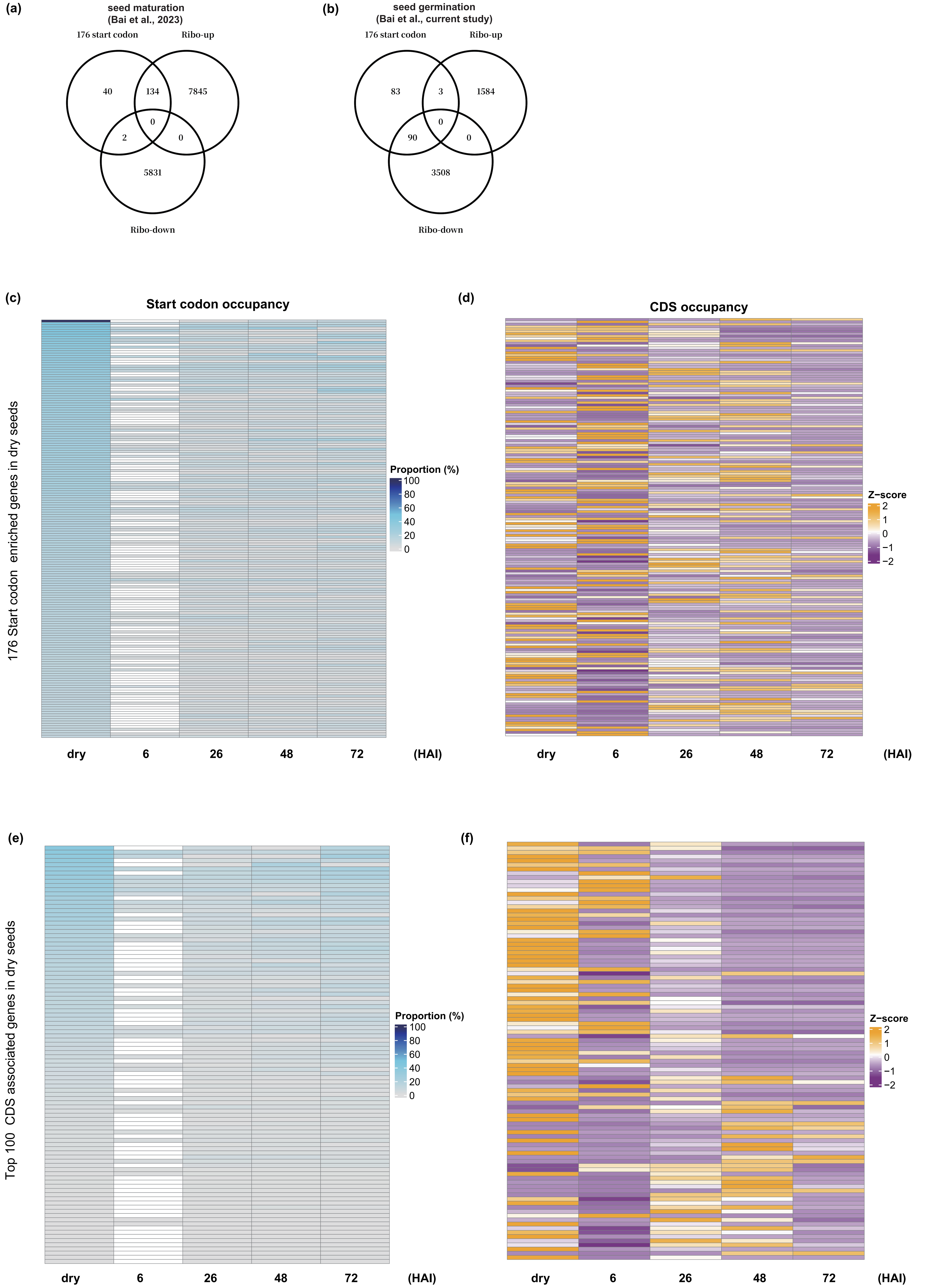

Figure S5. Investigation of the ribosome association of the 176 dry seeds start codon enriched genes during seed maturation and seed germination. (a) Venn diagram representing the overlap of the dry seed start codon enriched genes with polysome associated transcripts during seed maturation as retrieved from (Bai et al, 2023).
(b) Venn diagram representing the overlap of the start codon enriched genes with ribosome associated transcripts during seed germination that are retrieved in the current study. The transcripts indicated as Ribo-up or Ribo-down represent the sum of transcripts that have an increased or decreased ribosome association respectively.(c) Proportion of ribosome at the start codon for the 176 start codon enriched genes in dry seeds and (d) their corresponding CDS ribosome association during seed germination. (e) Proportion of ribosome at the start codon for the top 100 CDS ribosome associated genes in dry seeds and (f) their corresponding CDS ribosome association during seed germination.
